# Enhanced 3D Filter‐Exchange Imaging Using Multi‐Slab Acquisition

**DOI:** 10.1002/mrm.70593

**Published:** 2026-09-17

**Authors:** Jacopo Frignani, Athanasia Kaika, Ben Danzer, Franz Schilling

**Affiliations:** ^1^ Department of Nuclear Medicine, TUM School of Medicine and Health TUM University Hospital, Technical University of Munich Munich Germany; ^2^ CT‐MRI Department Hygeia Hospital Athens Greece; ^3^ Munich Institute of Biomedical Engineering Technical University of Munich Garching Germany

**Keywords:** 3D imaging, acceleration, AXR, FEXI, slab

## Abstract

**Purpose:**

Starting from the implementation of a single‐slice FEXI sequence, a 3D method was developed to accelerate a FEXI experiment and acquire a multi‐slice volume.

**Methods:**

3D multi‐slab FEXI divides the volume of interest into different slabs. In each slab, the slab‐selective diffusion and exchange modules are combined with slice‐selective detection modules. The 3D multi‐slab FEXI acquisition speed was assessed against single‐ and multi‐slice FEXI. In addition, the new method was compared to single‐slice FEXI on a yeast phantom.

**Results:**

3D multi‐slab FEXI acquires thirty slices within the same scan time required for single‐ and multi‐slice FEXI to acquire one and five slices, respectively. 3D multi‐slab FEXI found AXR values, homogeneity, repeatability, and reproducibility comparable to those of single‐slice FEXI, increasing SNR efficiency.

**Conclusion:**

3D multi‐slab FEXI accelerates a FEXI experiment and acquires a volume of several slices in the same scan time as the single‐slice FEXI protocol, giving comparable AXRs.

## 1 Introduction

DWI utilizes variations in the diffusion of water molecules in tissue to generate contrast [1, 2]. Water molecules experience diffusion not only within the intra‐ and extracellular compartments but also exchange across the cell membrane [3]. In particular, several channel proteins, such as aquaporins, facilitate osmotic water exchange, forming pores in the membrane, in addition to simple diffusion through the lipid bilayer membrane [4]. The expression of water transport channels, and consequently the water exchange across the cell membrane, is associated with several diseases [5, 6]. Therefore, imaging techniques that measure the water exchange across cell membranes can be used to diagnose pathologies and understand cell physiology [7, 8, 9]. Recently, AXR has been shown to be an early and sensitive biomarker of gradual cell membrane degradation [10, 11].

An example of these methods is FEXI, a non‐invasive imaging technique that measures water exchange rates across the cell membrane [12, 13]. FEXI acquires a signal after two diffusion‐weighted PGSE blocks that are separated by a variable delay period called the mixing time. The sequence is repeated several times, varying the mixing time and the b‐value of the second PGSE block to extract exchange information from the ADC recovery curve.

The necessity to acquire multiple ADCs, along with the requirements for numerous averages due to low SNR, given by the diffusion weighting, renders FEXI a time‐consuming sequence [12]. Therefore, repeating single‐slice FEXI several times on different slices to obtain a volume is not feasible for clinical applications.

The present work introduces a 3D version of FEXI, presented as 3D multi‐slab FEXI, which uses slab‐selective excitations to acquire several volumes of the sample in a single repetition. 3D multi‐slab FEXI accelerates a FEXI experiment and acquires a volume of several slices in the same scan time as the single‐slice FEXI method. This method is also faster than multi‐slice FEXI. 3D multi‐slab FEXI was evaluated on a yeast phantom and compared against the already established single‐slice FEXI [14, 15].

## 2 Methods

### 2.1 MRI Scanner

All measurements were conducted on a 7 T preclinical scanner (Discovery MR901 magnet and gradient system, Agilent, Santa Clara, CA, USA; and Avance III HD electronics, Bruker BioSpin MRI GmbH, Ettlingen, Germany). The software used as an interface was ParaVision 7.0.0. The measurements were performed using a dual‐tuned 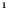
H/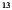
C volume resonator with a 31 mm inner diameter as the transceiver.

### 2.2 Phantom Preparation

3D multi‐slab FEXI was tested on a phantom containing a suspension of yeast cells [12, 16]. This yeast phantom consists of baker's yeast (Saccharomyces cerevisiae) that was dissolved in water at room temperature in a 2:1 ratio (yeast/water) and mixed with a magnetic stirrer for 20 min at moderate speed to obtain a homogeneous sample [14]. The mixture was then poured into a 50 mL Falcon tube and centrifuged at 1346 g for 20 min. Centrifugation creates a densely packed phantom, drastically speeding up the sedimentation process. After centrifugation, the water that settled on top of the pellet was discarded.

### 2.3 FEXI Pulse Sequence

#### 2.3.1 Single‐Slice FEXI

The entire method is a double PGSE sequence, in which the time between the two PGSE blocks is varied. The two diffusion‐sensitized spin‐echo elements are separated by an exchange module [12]. Every repetition contains a single‐slice FEXI block, shown in Figure 1a, which comprises three elements: the diffusion filter, the exchange module, and the detection module.

**FIGURE 1:**
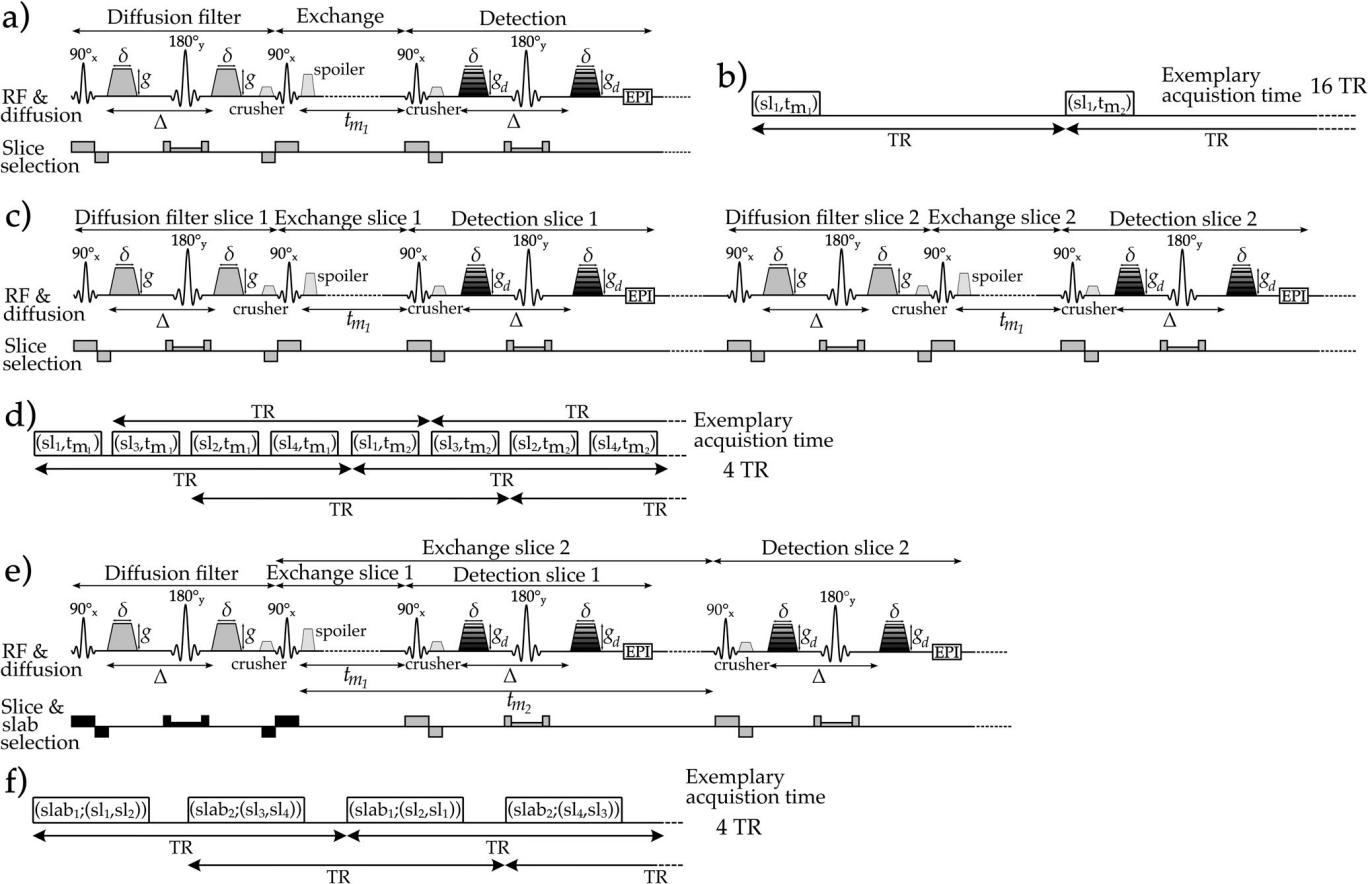
Pulse sequence diagrams and slice ordering comparison between single‐slice, multi‐slice, and 3D multi‐slab FEXI for four slices, two mixing times, and two detection b‐values. (a) Single‐slice FEXI block for one TR. The sequence comprises a slice‐selective single‐PGSE diffusion filter, followed by a slice‐selective longitudinal storage of the filtered magnetization where the exchange occurs. After the exchange module, the transverse magnetization is acquired by a detection module that combines a slice‐selective diffusion‐sensitized single‐PGSE and an EPI readout. Every RF pulse is slice‐selective. (b) Single‐slice FEXI contains one single‐slice FEXI block (sl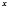, t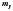) per repetition and has an acquisition time of 16 TR. In every TR, a different combination of slice, t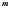, and detection b‐value is acquired. (c) Multi‐slice FEXI sequence for one TR. After the acquisition of the first slice, a second single‐slice FEXI block detects a second slice in the same TR. (d) In multi‐slice FEXI, several single‐slice FEXI blocks are included in one TR, using 4 TRs in total. The slice acquisition order is held constant to ensure that the TR is the same for each slice. After the first two TRs, the second detection b‐value is acquired. (e) 3D multi‐slab FEXI sequence for one slab within one TR. Rather than repeating the filter for each slice, a slab‐selective diffusion filter is applied once and is combined with many slice‐selective detection modules. In this way, the time for the exchange and the detection period of one slice are exploited for the exchange time of other slices too, saving time. The slab‐selective gradients are marked in black. (f) 3D multi‐slab FEXI needs 4 TRs in total. In every TR, slab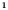 is acquired with a 3D slab FEXI block and slab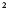 is measured with another block during the longitudinal relaxation of slab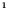. After the first two TRs, the second detection b‐value is acquired.

A diffusion‐weighted PGSE filter causes the dephasing of the magnetization of the fast‐diffusing water molecules, leading to greater signal suppression of the water molecules in the extracellular compartment and decreasing the measured ADC. The filter b‐value is constant in every repetition.

After suppression, the remaining phase of the transverse magnetization is stored in the longitudinal direction during the exchange module, during which water molecules exchange across the two intra‐ and extracellular compartments, leading to an ADC recovery. The variable period between the application of the filter and the acquisition is called the mixing time $t_{m}$.

In the detection module, the signal is acquired using single‐shot EPI after another diffusion‐weighted PGSE block. In contrast to the diffusion filter, the detection b‐value varies through repetitions to induce a diffusion‐weighting of the signal.

A spoiler gradient dephases the transverse magnetization generated by the excitation pulse of the exchange module. Two crusher gradients dephase the magnetization excited by the excitation pulse of the detection module.

The whole sequence consists of several repetitions in which the mixing time and detection b‐value vary. For example, consider the acquisition of four slices for two different mixing times and two detection b‐values. The total number of repetitions will be sixteen, as shown in Figure 1b.

#### 2.3.2 Multi‐Slice FEXI

Multi‐slice FEXI uses the same three modules as single‐slice FEXI, organizing the single‐slice FEXI blocks of different slices more efficiently [8, 17]. As illustrated in Figure 1c,d, multi‐slice FEXI uses the recovery period of the longitudinal relaxation of one slice to excite additional slices with other single‐slice FEXI blocks, ensuring equal TR for every slice throughout each repetition. The mixing time and detection b‐value vary across different repetitions. The acquisition slice order within a repetition is interleaved, and the slices are spaced by a gap to avoid cross‐talk introduced by interference between adjacent slices.

#### 2.3.3 3D Multi‐Slab FEXI

3D multi‐slab FEXI divides the object to be scanned into different volumes, also referred to as slabs. This new sequence is also based on the same three modules of the single‐slice FEXI method. However, the diffusion filter and the exchange module are applied to all slices of a slab simultaneously to obtain a signal at different mixing times for each slice in every repetition.

Figure 1e shows a 3D slab FEXI block for a two‐slice slab. First, the diffusion filter is applied to suppress the signal of fast‐diffusing water molecules in the entire slab. The RF pulses of the filter module are no longer slice‐selective but excite the entire slab. After the application of the filter, the magnetization of the slab containing both slices is stored in the longitudinal direction via slab‐selective excitation in the exchange module. During the longitudinal storage, the water molecules of the slab exchange between the two cellular compartments. After the first mixing time $t_{m_{1}}$, the signal from the first slice is acquired with the usual single‐slice selective detection module. During the first slice detection, the exchange continues in the second slice. When the longer mixing time $t_{m_{2}}$ is reached, the second slice magnetization is flipped in the transverse plane, and the detection module performs a signal acquisition.

This configuration is valid only if the mixing time $t_{m_{2}}$ of the second exchange period is sufficiently long to contain the exchange for $t_{m_{1}}$ and the detection modules. Hence, the following condition must hold

(1) $$t_{m_{2}}>t_{m_{1}}+t_{detection}$$

If Equation (1) is not fulfilled, the slab configuration cannot be used, reverting to the scheme used for multi‐slice FEXI.

A 3D slab FEXI block is applied to different slabs in every repetition, using the same idea as multi‐slice FEXI. For example, four slices and two mixing times can be divided into two slabs, slab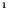 and slab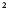. As illustrated in Figure 1f, slab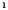 and slab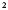 are excited and measured in every TR. This is possible because the spin dynamics within slab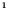 and slab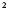 are independent.

### 2.4 Experimental Protocol

Several experiments were performed to validate and benchmark the proposed method against the already established single‐slice FEXI [14, 15]. In addition to an AXR comparison between single‐slice and 3D multi‐slab FEXI, three experiments were performed to test AXR homogeneity within each slice, repeatability, and reproducibility of 3D multi‐slab FEXI. Multi‐slice FEXI was not compared to single‐slice FEXI because it is slower than 3D multi‐slab FEXI and results in higher specific absorption rate and gradient demand.

All measurements were performed at a room temperature of $23^{\circ }$C. The FEXI protocol used the following parameters: number of slices: 6, slice thickness: 3 mm, slice gap: 1.8 mm, slab gap: 1.8 mm, TR: 4 s, TE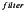: 25 ms, TE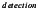: 25 ms, mixing times: 10, 100, 200 ms (comparable with value used in the already established single‐slice FEXI [14]), acquisition matrix size 42 × 42, FOV 64 × 64 mm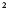, filter‐off repetitions: 16, filter‐on repetitions: 32, filter module b‐value: 1437 s/mm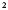, filter module diffusion gradient direction (0.71 $\hat{x}$, 0.71 ŷ, 0 $\hat{z}$), filter module gradient duration: 3 ms, filter module gradient separation: 8.9 ms, filter module spoiler amplitude: (0 $\hat{x}$, 0 ŷ, 177 $\hat{z}$) mT/m, detection module b‐values: 50, 600 s/mm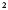, detection module diffusion gradient direction (0.71 $\hat{x}$, 0.71 ŷ, 0 $\hat{z}$), detection module gradient duration: 3 ms, detection module gradient separation: 8.9 ms, crusher amplitude: (118 $\hat{x}$, 0 ŷ, 0 $\hat{z}$) mT/m, spoiler amplitude: (118 $\hat{x}$, 118 ŷ, 0 $\hat{z}$) mT/m.

Before acquisition, B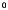 shimming was performed on the entire sample volume based on a B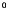 map. In addition, basic frequency, reference power, EPI trajectory, and receiver gain calibrations were performed. The receiver gain adjustment was repeated between the filter‐off and filter‐on measurements.

The single‐slice FEXI filter‐on acquired one slice in 12 min and 48 s. Therefore, the volume of six slices was acquired in 76 min and 48 s, repeating single‐slice FEXI on the different slices. The 3D multi‐slab FEXI filter‐on, including all mixing times, acquired the entire volume in 12 min and 48 s. The single‐slice FEXI filter‐off scan took 2 min and 8 s per slice, resulting in 12 min and 48 s for the volume. For the 3D multi‐slab FEXI filter‐off, the slices were interleaved using the multi‐slice FEXI approach since a single mixing time was acquired. The scan time resulted in 2 min and 8 s for the volume.

1. **Single‐slice and 3D multi‐slab FEXI AXR and SNR comparison.** The two methods were compared on the yeast cell phantom using the same protocol parameters. First, the single‐slice FEXI experiment was repeated six times to acquire the six slices of the volume. Then, 3D multi‐slab FEXI acquired the entire volume in a single acquisition, using two slabs of three slices.
2. **3D multi‐slab AXR homogeneity of a homogeneous phantom.** The AXR spatial homogeneity within a slice was evaluated for 3D multi‐slab FEXI. In addition, the homogeneity of AXR between slices was tested.
3. **3D multi‐slab FEXI repeatability.** The repeatability of the 3D multi‐slab FEXI sequence was tested on the yeast cell phantom. The method was repeated five times without removing the sample or re‐running the scanner adjustments.
4. **3D multi‐slab FEXI reproducibility.** The reproducibility of the 3D multi‐slab FEXI sequence was tested. Four 50 mL Falcon tubes were prepared from the same mixture of yeast and water. The samples were kept at the same temperature during the experiment. Each sample was scanned three times without repeating the adjustments. Between each sample, the adjustments were repeated.

### 2.5 Data Analysis

In each ROI voxel, the signals at different detection b‐values from the same slice and mixing time were fitted to obtain an ADC value. The different repetitions were not averaged before being fitted, but individually contributed to the fit cost function. In each voxel, the ADC values at different mixing times were fitted using Equation (2), where σ is the filter efficiency and $ADC^{eq}$ is the ADC without the signal suppression [15, 16].

(2) $$ADC(t_{m})=ADC^{eq}[1-\sigma e^{-t_{m}AXR}]$$

For each ROI, an AXR map was computed. The final AXR value and its error were the mean and standard deviation of the AXR map.

## 3 Results

### 3.1 Time‐Earning Performance

Given a defined TR, the maximum number of slices $n_{\max}$ that can be acquired per repetition was calculated for the different FEXI methods. Single‐slice FEXI has only one detection module per repetition regardless of the protocol.

(3) $$sFX:\;n_{\max}=1$$

Consider the acquisition of n slices at n different mixing times $t_{0},t_{1},\ldots ,t_{n-1}$ for multi‐slice (mFX) and 3D multi‐slab FEXI (slbFX). Equation (4) describes the condition that should be satisfied to acquire n slices at n different $t_{m}$.

(4) $$\begin{array}{cc} & mFX:\;TR-[n(t_{d}+t_{f})+n*t_{n-1}+t_{n-1}]>0\\ & slbFX:\;\frac{TR}{k}-(t_{d}+t_{f}+t_{n-1})>0 \end{array}$$

where $t_{d}$ and $t_{f}$ are the times needed for the detection module and the diffusion filter. The number of slabs that divide the volume of interest is referred to as k. In the multi‐slice case, the acquisition time is increased by the mixing time to ensure a constant recovery, as explained in Section 3.2. Since multi‐slice FEXI acquires a single $t_{m}$ per repetition, the longest mixing time $t_{n-1}$ must be used for Equation (4).

For 3D multi‐slab FEXI, the information encoding is most efficient when the number of mixing times is a multiple of the number of slices of a slab, and the mixing times satisfy Equation (1). In this case, each repetition contains k 3D slab FEXI blocks, increasing the number of readouts per repetition, thereby accelerating the sequence.

Assuming that the constant p equally spaces the mixing times ($t_{0}=0,\;\;t_{1}=p,\;\;\ldots ,\;\;t_{n}=n*p$). The maximum number of slices acquired per repetition, n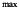, depends on the protocol. Considering a reasonable protocol (TR=3.1 s, p=100 ms, $t_{d}+t_{f}=50$ ms), multi‐slice FEXI can acquire five times more slices than single‐slice FEXI.

(5) $$mFX:\;n_{\max}=5$$

For 3D multi‐slab FEXI, $n_{\max}$ depends on the number of slabs k. Table 1 shows $n_{\max}$ and the number of slices per slab, considering different numbers of slabs. The number of slabs for the comparison was determined by ensuring that the same number of mixing times is acquired for both sequences. While multi‐slice FEXI can acquire 5 slices at 5 mixing times ($t_{0}=0,\;\ldots ,\;t_{n}=400$ ms) in a single repetition, 3D multi‐slab FEXI can acquire 30 slices per repetition using 5 mixing times, acquiring more information per repetition.

**TABLE 1:** 3D multi‐slab FEXI time performance. Given the number of slabs k, the maximum number of slices detected per slab n and the maximum number of detections per repetition n are shown together with the maximum mixing time t acquired. The results used the protocol parameters (TR=3.1 s, p=100 ms, $t_{d}+t_{f}=50$ ms).

k	n 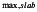	n 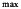	t 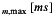
2	15	30	1400
3	10	30	900
4	8	32	700
5	6	30	500
**6**	**5**	**30**	**400**
7	4	28	300

### 3.2 3D Multi‐Slab T Bias

In single‐slice FEXI, the acquisition time per repetition is increased with the mixing time to ensure a constant recovery time across every mixing time. In 3D multi‐slab FEXI, it is not possible to avoid this T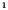 weighting since different mixing times are acquired in a single repetition. This T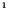 bias depends on the sample and the protocol, increasing with the difference between the first and last mixing times of a slab.

Considering that the yeast phantom has a T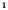 of 2 s, the T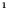 bias can be computed for the protocol used to compare 3D multi‐slab and single‐slice FEXI. Given a slice acquired at the mixing time $t_{m}$, the longitudinal net magnetization $M_{z}$ of the slice for the next repetition can be computed

(6) $$M_{z}=M_{0}(1-e^{-\frac{TR-t_{f}-t_{m}}{T_{1}}})$$

The maximum percentage difference in longitudinal magnetization recovery is always between the slices acquired with the shortest and longest mixing times within the slab and resulted in a percentage difference of 1.6%.

### 3.3 Single‐Slice and 3D Multi‐Slab FEXI AXR Comparison

An exemplary diffusion‐weighted image is shown for single‐slice and 3D multi‐slab FEXI in Figure 2a,e. In addition, an AXR map is illustrated for the two sequences in Figure 2b,f. The two methods yielded equivalent AXRs, as shown in Figure 2c. The mean and standard deviation across the slices were computed for both methods (AXR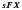 = 1.35 ± 0.05 s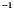, AXR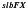 = 1.39 ± 0.06 s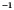). The AXR coefficient of variation (CV = standard deviation/mean) between different slices is similar in the different methods (CV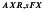 = 3.5%, CV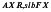 = 4.1%). In addition, the homogeneity within each slice was compared for the two methods. The AXR CVs for single‐slice FEXI, CV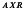 = (0.15, 0.19, 0.15, 0.15, 0.16, 0.14), corresponding to slices 1 through 6, are comparable with the values of 3D multi‐slab FEXI, CV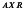 = (0.19, 0.16, 0.17, 0.15, 0.17, 0.17).

**FIGURE 2:**
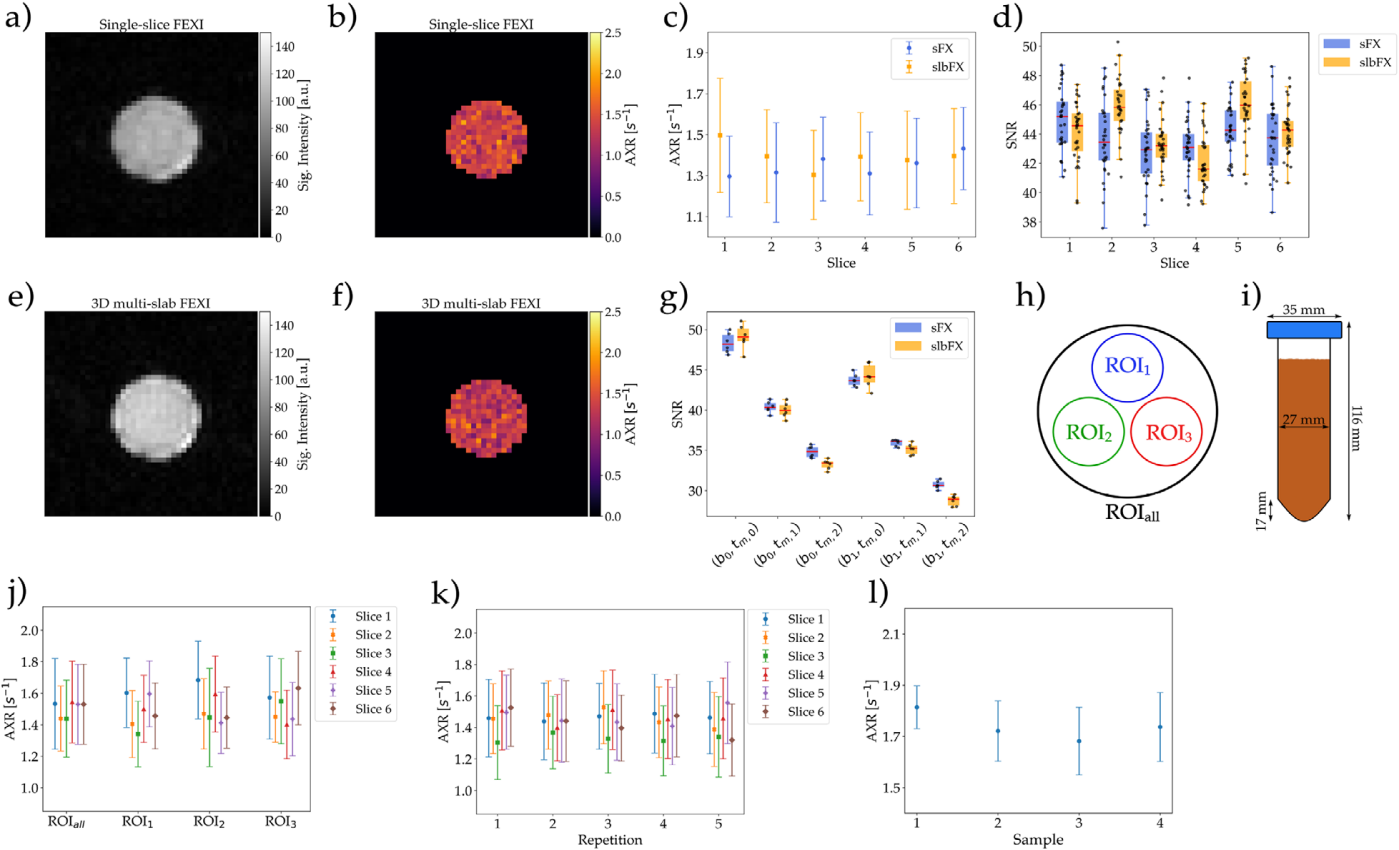
(a) Single‐slice FEXI exemplary diffusion‐weighted image. The image shows the filter‐on signal acquired from the third slice, first repetition, first mixing time, and first detection b‐value. (b) Single‐slice FEXI exemplary AXR map. The image illustrates the map for the third slice of the phantom. (c) AXR comparison between single‐slice (sFX) and 3D multi‐slab FEXI (slbFX). For every slice, the mean and standard deviation of the AXR map within ROI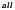 are displayed. (d) Exemplary SNR comparison between single‐slice and 3D multi‐slab FEXI. The image illustrates the SNR values of all the different repetitions for the first detection b‐value and mixing time in the different slices. (e) 3D multi‐slab FEXI exemplary diffusion‐weighted image. The image shows the filter‐on signal acquired from the third slice, first repetition, first mixing time, and first detection b‐value. (f) 3D multi‐slab FEXI exemplary AXR map. The image illustrates the map for the third slice of the phantom. (g) Averaged SNR comparison between single‐slice and 3D multi‐slab FEXI. For every slice, mixing time, and detection b‐value, the SNR values of the different repetitions were averaged, and the results are compared between the two sequences. Every box contains different slices of the phantom. (h) Geometry of different ROIs. ROI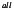 contains the entire axial phantom section. ROI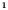, ROI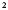, ROI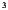 were drawn inside the phantom. (i) Graphical representation of a 50 mL Falcon tube filled with yeast. (j) 3D multi‐slab AXR homogeneity. For each slice, the mean and standard deviation of the AXR map within ROI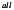 were computed in four separate regions (ROI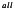, ROI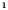, ROI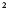, ROI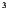). (k) 3D multi‐slab FEXI repeatability. For each slice, the mean and standard deviation of the AXR map within ROI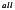 are illustrated in the five different repetitions of the experiment. (l) 3D multi‐slab reproducibility. For each sample, the AXR mean and standard deviation for every slice and experiment repetition within ROI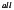 are displayed.

### 3.4 Single‐Slice and 3D Multi‐Slab FEXI SNR Comparison

As described in Section 2.5, the different repetitions are not averaged in the complex space to compute the final AXR; therefore, the SNR was compared between the sequences for each repetition individually. Figure 2d shows an exemplary SNR comparison between single‐slice and 3D multi‐slab FEXI for the first detection b‐value and mixing time $t_{m,1}$. In addition, the SNR values from the different repetitions were averaged for every slice, mixing time, and detection b‐value. While SNR is comparable between sequences at the initial mixing time, the 3D multi‐slab approach suffers an SNR drop at longer mixing times, as illustrated in Figure 2g. This drop may be primarily attributed to the RF pulses from the detection modules of previously detected slices, which act as magnetization transfer pulses during the exchange of subsequent slices and partially saturate their signal. The SNR percentage difference between the two sequences was computed at different $t_{m}$ and detection b‐values after averaging the SNR of different slices and never exceeded 1.7%. Furthermore, the AXR values proved to be comparable between the single‐slice and 3D multi‐slab approaches, despite the minor signal reduction at extended mixing times, as shown in Section 3.3.

The SNR efficiency was computed for the sequences, averaging the SNR over all measurements (repetition, mixing time, and detection b‐value) and dividing it by the square root of the total scan time to acquire the six slices. 3D multi‐slab FEXI yielded an SNR efficiency of 1.4 $s^{-1/2}$ against the 0.6 $s^{-1/2}$ of the single‐slice method, representing a 133% increase.

### 3.5 3D Multi‐Slab FEXI Homogeneity

In addition to ROI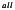, the AXR was computed in the other three ROIs, illustrated in Figure 2h. As shown in Figure 2j, the AXRs are comparable across the different ROIs. Moreover, the AXR variation within every slice of ROI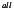 is similar: CV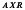 = (0.03, 0.02, 0.05, 0.05, 0.05, 0.05).

### 3.6 3D Multi‐Slab FEXI Repeatability

The AXRs of each slice are shown for each repetition in Figure 2k. For each slice, the CV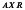 across the experiment repetitions is illustrated in Table 2. The coefficient of variation values align with those measured for single‐slice FEXI in the literature [14].

**TABLE 2:** Repeatability results. For each slice, the average AXR across the five repetitions is shown with its standard deviation. In addition, the AXR CV is reported.

	Slice 1	Slice 2	Slice 3	Slice 4	Slice 5	Slice 6
AXR $\left[s^{-1}\right]$	1.46	1.46	1.33	1.47	1.47	1.43
$\sigma_{AXR}$ $\left[s^{-1}\right]$	0.02	0.05	0.02	0.04	0.05	0.07
CV 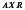 [%]	1.1	3.2	1.7	2.8	3.6	4.9

### 3.7 3D Multi‐Slab FEXI Reproducibility

For each slice, the AXRs of each sample and repetition were averaged. The results are depicted in Table 3. The CV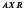 was comparable to those reported in the literature [14]. The AXRs of different repetitions and slices were combined for each sample. The results are illustrated in Figure 2l.

**TABLE 3:** Reproducibility results. For each slice, the average AXR across each sample and repetition is shown with its standard deviation. In addition, the AXR CV is reported.

	Slice 1	Slice 2	Slice 3	Slice 4	Slice 5	Slice 6
AXR $\left[s^{-1}\right]$	1.82	1.70	1.61	1.79	1.76	1.74
$\sigma_{AXR}$ $\left[s^{-1}\right]$	0.10	0.11	0.17	0.09	0.08	0.08
CV 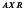 [%]	5.7	6.2	10.1	4.9	4.3	4.6

## 4 Discussion

The 3D slab FEXI sequence presented is more efficient in encoding information than single‐slice and multi‐slice FEXI, allowing for reductions in total scan time. The drastic increase in the possible number of slices using 3D multi‐slab FEXI makes the sequence promising for in vivo applications, as a shorter scanning time results in less stress for the patient or animal and mitigates the risk of motion artifacts. In addition, shorter scan times will allow for a higher throughput, increasing accessibility [18]. Alternatively, instead of acquiring a larger volume, 3D multi‐slab FEXI can acquire the same number of slices as those obtained using many repetitions of single‐slice FEXI on different slices while increasing the SNR efficiency, either through a lower scan time or by acquiring more averages in the same scan time.

As reported by Lasič, imaging and slice selection gradients impart an additional diffusion weighting during the longitudinal storage interval, leading to an AXR underestimation [19, 20]. In 3D multi‐slab FEXI, the RF pulses contained in the diffusion and exchange modules excite an entire slab rather than a single slice. This is achieved by reducing the slab‐selective gradient amplitudes. Therefore, the additional diffusion weighting contribution was smaller, reducing the AXR underestimation. Nevertheless, 3D multi‐slab FEXI gave results comparable to single‐slice FEXI in the yeast sample, since the large slice thickness employed minimized the AXR underestimation effect [19].

In addition to the techniques presented, the FEXI acquisition could be accelerated by improving the AXR map through denoising techniques, reducing the number of repetitions [21].

Moreover, the new method also proved to be repeatable and reproducible, giving similar AXR performance to single‐slice FEXI and increasing the SNR efficiency. In addition, the T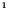 bias introduced by acquiring multiple mixing times in a single repetition had a negligible impact on the AXR computation. These results make 3D multi‐slab FEXI a good candidate when the acquisition of a large volume composed of several slices or high SNR efficiency is needed for a FEXI examination.

## 5 Conclusions

Starting from a single‐slice FEXI sequence, a 3D FEXI method, introduced as 3D multi‐slab FEXI, was implemented to speed up a FEXI examination. Depending on the protocol, 3D multi‐slab FEXI acquires thirty slices within the same scan time required for single‐ and multi‐slice FEXI to acquire one and five slices, respectively, obtaining more information. Lastly, 3D multi‐slab FEXI was evaluated against the already established single‐slice FEXI on a yeast phantom. The AXRs were comparable to those obtained for single‐slice FEXI, and SNR efficiency was higher. Moreover, the new sequence was proved to be repeatable and reproducible.

## Funding

The present work was supported by the Deutsche Forschungsgemeinschaft (DFG) (Grant Nos. 391523415, SFB 824) and the Young Academy of the Bayerische Akademie der Wissenschaften.

## Conflicts of Interest

The authors declare no conflicts of interest.

## Data Availability

The data that support the findings of this study are available from the corresponding author upon reasonable request.
